# The Function of Thioredoxin-Binding Protein-2 (TBP-2) in Different Diseases

**DOI:** 10.1155/2018/4582130

**Published:** 2018-05-02

**Authors:** Jianghua Hu, Yibo Yu

**Affiliations:** Eye Center, Second Affiliated Hospital, School of Medicine, Zhejiang University, Hangzhou, Zhejiang, China

## Abstract

Thioredoxin-binding protein-2 (TBP-2) has an important role in the redox system, but it plays a different role in many different diseases (e.g., various cancers, diabetes mellitus (DM), cardiovascular disease, and cataracts) by influencing cell proliferation, differentiation, apoptosis, autophagy, and metabolism. Distinct transcription factors (TFs) stimulated by different factors combine with binding sites or proteins to upregulate or downregulate TBP-2 expression, in order to respond to the change in the internal environment. Most research disclosed that the main function of TBP-2 is associating with thioredoxin (Trx) to inhibit the antioxidant capacity of Trx. Furthermore, the TBP-2 located in tissues, whether normal or abnormal, has the ability to cause the dysfunctioning of cells and even death through different pathways, such as shortening the cell cycle and inducing apoptosis or autophagy. Through these studies, we found that TBP-2 promoted the development of diseases which are involved in inflammatory and oxidative damage. To a certain extent, we believe that there is some hidden connection between the biological functions which TBP-2 participates in and some distinct diseases. This review presents only a summary of the roles that TBP-2 plays in cancer, DM, cataracts, and so on, as well as its universal mechanisms. Further investigations are needed for the cell signaling pathways of the effects caused by TBP-2. A greater understanding of the mechanisms of TBP-2 could produce potential new targets for the treatment of diseases, including cancer and diabetes, cardiovascular disease, and cataracts.

## 1. Introduction

In recent years, as thioredoxin-binding protein-2 (TBP-2) has been investigated more and more, the significance of its functions has become more apparent. It is closely related not only to cellular processes such as proliferation, differentiation, and apoptosis, but also to metabolism, gene expression, and redox reactions. Many studies have found that TBP-2 is overexpressed in patients with T2DM (type II diabetes mellitus) [1], cardiovascular diseases [2], and cataracts [3] and many studies have also shown that TBP-2 expression is decreased in tumor cells [4], such as those caused by colorectal and gastric cancers [5].

TBP-2 was originally identified as a protein that is upregulated in human promyelocytic leukemia cells (HL60), which are treated with 1,25-(OH)_2_D_3_; therefore, TBP-2 was initially called “vitamin D_3_ upregulated protein 1” [6]. Then, by using the yeast two-hybrid system, Nishiyama et al. [7] found that TBP-2 is a thioredoxin- (Trx-) binding protein and demonstrated that it binds to the catalytic active center of Trx (instead of oxidized Trx) to inhibit Trx activity and expression. And soon afterwards, TXNIP is reported by Bodnar's group, and they identified TBP-2 as a thioredoxin-interacting protein (TXNIP) [8]. Since then, many studies have discovered that TBP-2 can be regulated by mechanical stress, UV light, heat shock, hypoxia, H_2_O_2_, NO, glucose, and insulin [9–17].

## 2. TBP-2 Genomic Organization

The human TBP-2 gene has a nucleotide sequence that is 77% similar to that of zebrafish and 89% similar to that of mice, meaning that the TBP-2 gene is highly conserved [6]. This is strong evidence that TBP-2 has basic and vital biological functions. The gene is located on chromosome 1q21.1; it contains 8 exons and 7 introns and is 4174 bp in length. The TBP-2 promoter contains a carbohydrate response element (ChoRE), which commands TBP-2 expression under glucose responsiveness [18], as well as CCAAT and TATA sequences. Downstream of the gene, 1.3 kb consensus sites for polyadenylation have been identified as an untranslated 3′ region [19].

TBP-2 consists of 391 amino acids (AAs), and its molecular weight is 46 kDa. TBP-2 belongs to the *α*-arrestin family and is the only family member presently known to bind to Trx [20]. It contains an arrestin-like N-terminus (10–152 AAs) and an arrestin-like C-terminus (175–298 AAs).

The Trx system consists of NADPH, thioredoxin reductase (TrxR), and Trx; it is one of the crucial redox systems. Trx is a ubiquitous, multifunctional, 12 kDa protein found in all living cells, and it has two major protein isoforms that differ in subcellular structure: cytosolic Trx1 and mitochondrial Trx2. Their active sites in Trx are -Cys-Gly-Pro-Cys-; in order to preserve the intracellular redox balance, the two cysteine residues of Trx (Cys32 and Cys35) undergo reversible oxidation-reduction reactions [21].

Oxidized TBP-2 contains a disulfide linkage between Cys-63 and Cys-247, and the oxidized TBP-2's Cys-247 reacts with Trx's Cys-32 to form a disulfide. A gene mutation in the Cys-247 of TBP-2 is sufficient to abolish its inhibitory function of Trx activity [22]. It has been suggested that the two intramolecular disulfide bonds between the Cys-63 and the Cys-247 of TBP-2 are crucial for the efficient interaction of TBP-2 with Trx. Moreover, there are two PPXY motifs (331–334 AAs and 375–378 AAs) at the C-terminus that are known to interact with WW domains; they may participate in regulating TBP-2 protein turnover, as they can directly interact with E3 ubiquitin ligase ITCH, promoting proteasome degradation of TBP-2 [23].

## 3. TBP-2 Expression

Apart from vitamin D3, which can regulate transcription of TBP-2 [6], a large number of stimuli regulate TBP-2 expression, including mechanical stress, fluid shear stress, UV light, heat shock, hypoxia, H_2_O_2_, NO, nicotinamide adenine dinucleotide (NADH), ATP, glutamine, nicotine, vascular endothelial growth factor, basic fibroblast growth factor, transforming growth factor *β* (TGF-*β*), estradiol, calcium channel blockers, activation of receptors for advanced glycation end products (RAGE), insulin, and glucose [6, 10–17, 21, 24–28]. Anticancer agents such as 5-fluorouracil, anisomycin, dexamethasone, and ceramide also dramatically induce TBP-2 expression [29].

According to past studies [30], the TBP-2 promoter contains a ChoRE, a CCAAT box, and a TATA box. There are three main transcription factors (TFs): the heterodimeric TF MondoA:Max-like protein X (Mlx), the trimeric TF nuclear factor Y (NF-Y), and the MondoA paralog carbohydrate response element-binding protein (ChREBP). ChREBP, which is induced by glucose, can upregulate TBP-2 expression through the ChoRE [4, 31]. Under glucose stimulation, the more NF-Y binds to the CCAAT boxes, the more MondoA:Mlx moves from the outer mitochondrial membrane to the cell nucleus to integrate with the ChoRE; this can directly activate the TBP-2 promoter [30, 32]. Both NF-Y and MondoA:Mlx synergistically activate TBP-2 transcription. Furthermore, MondoA:Mlx not only plays a role in the upregulation of TBP-2 under glucose but is also involved in regulating TBP-2 expression in response to various other metabolic parameters, such as hypoxia [33], lactic acidosis [34], inhibition of oxidative phosphorylation [35], and the presence of molecules that contain adenosine—for example, NADH or ATP [28].

Other studies have also examined other TFs. Studies that investigated the forkhead box O1 (FOXO1) and FOXO3A showed that FOXO1 is associated with the TBP-2 promoter at the FOXO binding site and to increased TBP-2 expression [36, 37]. Another TF is peroxisome proliferator-activated receptor gamma (PPAR*γ*) [38]. In addition, other binding sites of TFs are AP-1, ETS1, the glucocorticoid receptor, KLF6, and others; they can all promote TBP-2 expression.

Epigenetic modification is another mechanism of transcriptional control, as in the methylation of a regulatory promoter region. One study discovered that DNA methylation at CpG sites in the promoter and exon 1 and histone deacetylation all induce TBP-2 expression to be silent. Other specific factors that regulate TBP-2 expression through interactions with its promoter are a heat shock factor binding to a heat shock element [39], polycomb repressive complex 2 [40], and heterogeneous nuclear ribonucleoprotein G [41].

## 4. TBP-2 Function

In all parts of our lives, from embryonic development to biological activity, precisely regulated cell differentiation and proliferation keep our bodily operations normal. TBP-2 plays a vital role in the development and differentiation of a variety of different tissues and cell types. As for its distribution, TBP-2 is found in brain tissues, the adrenal gland, cardiac tissues, lung tissues, the spleen, testes, skeletal muscles, kidney, liver, mammary glands, ovaries, and elsewhere. Interestingly, it is relatively highly expressed in lung tissues and less expressed in the brain and liver tissues [29]. TBP-2 mainly exists in the cytoplasm and the cell nucleus; however, intracellular distribution is different in different conditions. TBP-2 is located in the cytoplasm and the cell nucleus when there is a high cell concentration; otherwise, it is mainly in the cell nucleus [42].

### 4.1. TBP-2 in Cancer

TBP-2 suppresses cellular proliferation and arrests the cell cycle; it has also been reported to be a tumor suppressor gene [43, 44]. According to current research, the downregulated expression of TBP-2 is found in various kinds of human cancer cells, such as those from breast cancer, colon cancer, prostate cancer, bladder cancer, gastrointestinal cancer, lung cancer, malignant pheochromocytomas, high-grade B cell lymphoma, and adult T-cell leukemia (ATL) [4, 5, 42, 45, 46], although TBP-2 was highly expressed in these normal tissues.

The expression of TBP-2 in cancer decreases in two pathways. First of all, when human T-cell leukemia virus type I (HTLV-I) infects T-cells, it results in adult T-cell leukemia, and the T-cell growth form transitions from interleukin- (IL-) 2-dependent to IL-2-independent [47]. This transition is accompanied by a loss of TBP-2 expression that is induced by DNA methylation at CpG sites in the promoter and exon 1 and histone deacetylation. Another one is that HDAC1 can be recruited to the TBP-2 promoter to decrease TBP-2 expression by forming a protein complex with the RET finger protein (RFP) and NF-Y [48]. An anticancer drug, suberoylanilide hydroxamic acid (SAHA), can also inhibit HDAC1 and induces upregulation of TBP-2 expression through the inverted CCAAT box in the TBP-2 promoter, so that it leads to a block in growth arrest [4].

With the above approaches, cell growth is uncontrolled. On the contrary, tumor cells that are transfected with overexpressed TBP-2 show slowed cell growth. Overexpressed TBP-2 combined with Jun activating binding protein 1 (JAB1) with carboxyl (-COOH) restrains the translocation of p27^kip1^, a cyclin-dependent kinase (CDK) inhibitor that evidently has reduced expression in tumor cells, from the nucleus to the cytoplasm. This results in increasing p27^kip1^ stability in the nucleus, whereby p27^kip1^ can inhibit the CDK system of cyclin A to arrest the transition from the G_1_ to the S phase [49]. On the other hand, overexpressed TBP-2 combined with another corepressor complex, including promyelocytic leukemia zinc-finger (PLZF), Fanconi anemia zinc-finger (FAZF), and histone deacetylase 1, arrest cell cycle at the G_0_/G_1_ phase and suppress cyclin A2 promoter activity and the IL-3 receptor [42]. These suggests that TBP-2 controls tumor-suppression activity.

In addition, macrophage migration inhibitory factor (MIF), a cytokine overexpressed in various tumors, can regulate the immune response and tumorigenesis under inflammatory conditions. In cervical cancer, MIF combines with TBP-2 to ultimately break the negative regulation of TBP-2 on the NF-*κ*B pathway and enhances cell proliferation and migration leading to the progression of cancer [50].

**Figure 1:**
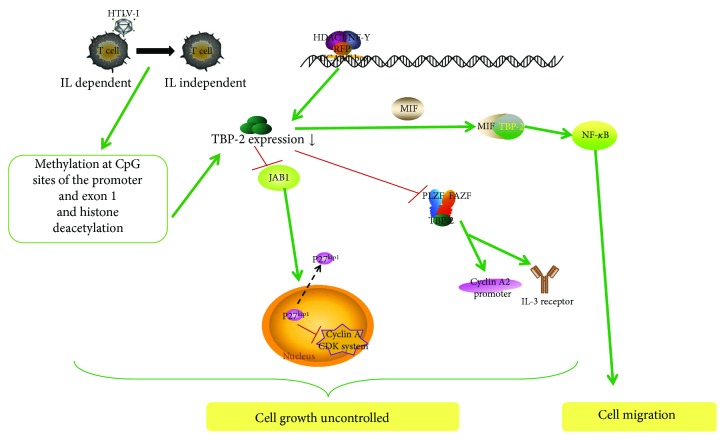
TBP-2 suppresses cell growth in cancer disease. TBP-2 expression can be decreased by DNA methylation at CpG sites, histone deacetylation, and the complex of HDAC1, RFP, and NF-Y. Low expression of TBP-2 could not combine with JAB1 to restrain the translocation of p27^kip1^ induced by JAB1; this results in increased p27^kip1^ stability in the cytoplasm, whereby p27^kip1^ cannot inhibit cell cycle procession. Meantime low TBP-2 cannot give assistance to the corepressor complex of PLZF, FAZF, and histone deacetylase 1 to induce cell cycle arrest. Moreover, when TBP-2 interacts with MIF, it could not suppress NF-*κ*B activity since cancer cells migrate.

In summary, these findings provide strong evidence that TBP-2 plays a vital role in cell cycle regulation and cancer biology. TBP-2 not only acts as an antitumor gene that can suppress cell growth at the G_0_/G_1_ and G_1_/S phase transitions in different cells by forming different transcriptional repressor complexes, but also acts as an inhibitor to suppress the migration of cancer cell (Figure 1).

### 4.2. TBP-2 in DM

#### 4.2.1. TBP-2 in Common Diabetes

TBP-2 acts as a gene that is highly responsive to blood glucose levels and insulin signaling and is overexpressed in the skeletal muscles of patients with impaired glucose tolerance or T2DM [1]. It is suppressed by insulin in human muscles and adipocytes, and it is elevated under glucose treatment [1]; this indicates that the expression of TBP-2 is mutually beneficially regulated by insulin and glucose. Under glucose stimulation, on the one hand, the more NF-Y binds to the CCAAT boxes, the more MondoA:Mlx moves from the outer mitochondrial membrane to the cell nucleus to integrate with the ChoRE, where it can directly activate the TBP-2 promoter [30, 32]. On the other hand, FOXO1 controls an alternative transcriptional expression of TBP-2 via p38 MAPK (mitogen activated protein kinase) [36]. Lastly, PPAR*γ*, which controls genes involved in fatty acid and glucose metabolism, can elevate the expression of TBP-2, and, in turn, overexpressed TBP-2 negatively regulates PPAR*γ* activity [38]. This reciprocal feedback mechanism underlines the role that TBP-2 plays in glucose metabolism and lipid metabolism. In accordance with these three pathways, TBP-2 is overexpressed in response to glucose. In contrast, insulin downregulates TBP-2 expression; this requires insulin receptor signaling instead of a simple reduction in glucose concentration [1].

Overexpressed TBP-2 impairs glucose metabolism [18, 51]. However, many hypoglycemic people show reduced TBP-2 expression [15, 52, 53]. Obesity, which produces adipocytokines, is involved in insulin resistance and glucose intolerance. Thus, when obesity, free fatty acids, and high glucose upregulate TBP-2 expression, this progress inhibits the expression of several insulin signaling-related genes, such as insulin receptor substrate-1 (IRS-1) [54], and Akt phosphorylation [55]. As IRS-1 protein levels decrease and Akt is not phosphorylated, insulin sensitivity is weakened. It should be mentioned that all of these effects occur only in muscle tissue.

A deficiency of TBP-2 enhances glucose-stimulated insulin secretion (GSIS) and mitochondrial ATP production in pancreatic *β*-cells, while upregulated TBP-2 restrains them [54]. Mitochondrial uncoupling protein-2 (UCP-2) has a negative effect on GSIS and glucose-induced ATP [56]. TBP-2 upregulates the expression and transcriptional activity of UCP-2 by recruiting PGC-1*α*, whose increasing activity could enhance the expression of UCP-2 to the UCP-2 promoter [54, 57]. Consequently, TBP-2 indirectly inhibits insulin secretion by raising the transcriptional activity of UCP-2.

**Figure 2:**
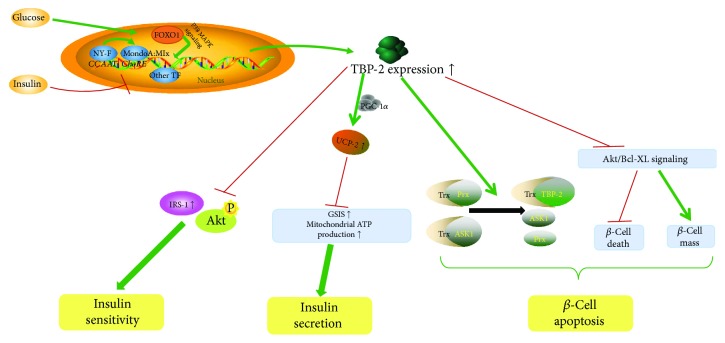
TBP-2 reduces insulin sensitivity, inhibits the secretion of insulin, and accelerates *β*-cell apoptosis in common diabetes. NF-Y, MondoA:Mlx, FOXO1, and other TFs can activate the TBP-2 promoter to upregulate its expression under glucose stimulation. The overexpression of TBP-2 inhibits the expression of IRS-1 and Akt phosphorylation to weaken insulin sensitivity, indirectly suppresses insulin secretion by raising the transcriptional activity of UCP-2, and increases the rate of pancreatic *β*-cell apoptosis by competing with ASK1 to bind with Trx and inactivating Akt/Bcl-xL signaling.

TBP-2 is an important regulator of pancreatic *β*-cell apoptosis [18, 58]. TBP-2 can compete with peroxiredoxin (Prx) and apoptosis signal-regulating kinase 1 (ASK1) to bind with Trx, which impairs Trx's function and induces pancreatic *β*-cell apoptosis [59]. Trx plays a vital role in protecting cells from oxidative stress and apoptosis, so it can prevent the progression of T2DM under oxidative stress which is induced by glucose in *β*-cells [60, 61]. As pancreatic *β*-cell loss through apoptosis increases, diabetes mellitus is aggravated. Conversely, TBP-2 deficiency activates Akt/Bcl-xL signaling, and this activity inhibits mitochondrial *β*-cell death and increases the endogenous *β*-cell mass [62]. Therefore, it is suspected that overexpressed TBP-2 may also promote *β*-cell apoptosis by inhibiting Trx under oxidative stress (Figure 2).

#### 4.2.2. TBP-2 in DM Complications

There are many microvascular complications in DM, such as nephropathy, neuropathy, and retinopathy. As is well known, chronic high glucose (HG) and an increase in receptors of advanced glycation end products (RAGE) facilitate the development of DM complications.

**Figure 3:**
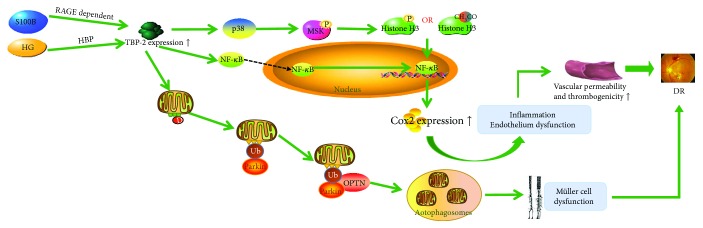
TBP-2 induces inflammation and endothelium dysfunction by the p38 MAPK-NF-*κ*B signaling pathway. S100B and HG induce TBP-2 to overexpress. Through the p38 MAPK-NF-*κ*B signaling pathway and modifications of histone H3 lysine K9, overexpressed TBP-2 brings about inflammation and endothelium dysfunction. On the other hand, the activation of the TXNIP-Drp1-Parkin-OPTN (p62/SQSTM1) axis may heighten mitophagy, which will ultimately lead to the reduction of mitochondrial number. In the end, these conditions lead to diabetic retinopathy.

In diabetic retinopathy, S100B stimulation induces RAGE-dependent TBP-2 expression, and HG induces TBP-2 via the hexosamine biosynthesis pathway (HBP). Overexpressed TBP-2 induces inflammation through the p38 MAPK-NF-*κ*B signaling pathway and modifications of histone H3 lysine K9. The activation of p38 induces the modification of the chromatin structure and increases the DNA binding of NF-*κ*B via the activation of downstream nuclear mitogen- and stress-activated protein kinase (MSK) to phosphorylate histone H3. Phosphorylation of histone H3 induces Cox2 expression, which leads to ocular inflammation and endothelial dysfunction [13]. Moreover, overexpressed TBP-2 could promote mitophagy in rat Müller cell line (rMC1) under HG conditions. TBP-2 and ROS/RNS stress, which is caused by TBP-2 itself, leads to mitochondrial dysfunction and damage through the TXNIP-Drp1-Parkin-OPTN (p62/SQSTM1) axis. In this pathway, Fis1 on the impaired mitochondrial outer membrane binds to Drp1 and arrests Drp1 on the mitochondrial membrane, thereupon, the E3 ubiquitin ligase Parkin is recruited to interact with ubiquitinated mitochondrial membrane proteins, such as VDAC1 and fusion proteins Mfn2. Subsequently, mitochondria bind to the ubiquitin receptor OPTN via Parkin and target the damaged mitochondria to autophagosomes. Then, the lysosome fuses with the autophagosome, which in turn dissolves the mitochondria [63]. These conditions cause increasing vascular permeability and thrombogenicities, resulting in diabetic retinopathy (Figure 3).

**Figure 4:**
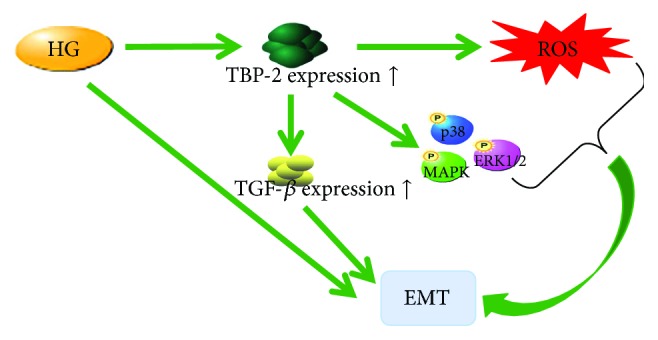
TBP-2 induces EMT. EMT was facilitated by TBP-2 via augmentation by ROS, the phosphorylation of p38 MAPK and ERK1/2, and the overexpression of TGF-*β*1.

Diabetic nephropathy is not only a complication of DM, but also one of the causes of chronic kidney disease (CKD), the severity of which relies on tubulointerstitial fibrosis, which is associated with the epithelial-to-mesenchymal transition (EMT) induced by high glucose and TGF-*β*. One study showed that the knockdown of TBP-2 inhibits EMT via the suppression of reactive oxygen species (ROS), the phosphorylation of p38 MAPK and ERK1/2, and the overexpression of TGF-*β*1 [64] (Figure 4). In addition, it is known that the overexpression of TBP-2 induced by HG contributes to the dysfunction of autophagy in renal proximal tubular cells (PTCs) under diabetic conditions, so that autophagic flux is impaired, and PTCs undergo apoptosis [65]. However, the concrete molecular mechanisms of these processes are unclear (Figure 4).

Briefly, under high levels of free fatty acids or glucose, the expression of TBP-2 is upregulated, bringing about a reduction in insulin sensitivity, the secretion of insulin, and the facilitation of *β*-cell apoptosis. Also, the complications of DM are due to the overexpression of TBP-2, which is caused by hyperglycemia that gives rise to inflammation, autophagy, and the promotion of the formation of EMT in the capillaries. These findings hint that *β*-cells, PTCs, or microvascular endothelial cells that express low TBP-2 could be part of novel strategies for the treatment of diabetes.

### 4.3. TBP-2 in Cardiovascular Disease

It is known that many factors contribute to diseases of the cardiovascular system; for instance, oxidative stress and fluid shear stress are common factors. Oxidative stress induced by hyperglycemia in the heart results in apoptosis and, ultimately, cardiomyopathy. Regions of the cardiovascular system that are exposed to disturbed flow are susceptible to atherosclerosis [66].

As the antioxidant transcription factor, nuclear factor erythroid 2-related factor 2 (Nrf2) is activated by a range of oxidants and electrophiles in cardiovascular disease. Upon dissociation from its cytosolic adaptor protein Keap1, Nrf2 accumulates in the nucleus and activates a wide range of genes that encode antioxidant proteins. Via binding to the antioxidant response element of the TBP-2 promoter, Nrf2 negatively controls both basal and stimulated TBP-2 expression [67].

Under oxidative stress, the major effects of TBP-2 are relevant to inflammation and apoptosis. First, TBP-2 expression is directly increased by a superoxide (O_2_^−^) that is induced by NADPH oxidase [68] and glucose. Afterwards, overexpressed TBP-2 results in the activation of the endothelial nod-like receptor protein 3 (NLRP3) inflammasome and several downstream inflammatory mediators, including IL-1*β*. Similar mechanisms have been demonstrated in a rat model in which a high-fat diet was accompanied by TBP-2 release and inflammatory activation [69]. Second, increased TBP-2 can cause apoptosis via the disruption of the Trx-ASK1 complex. TBP-2 competes with ASK1 to bind with Trx [59]. When TBP-2 binds with Trx, it breaks the Trx-ASK1 complex; ASK1 is then phosphorylated to its active form, inducing apoptosis through the activation of the JNK and p38 cascades [70], thus arresting the cell cycle [71].

Additionally, under fluid shear stress, when endothelial cells (ECs) are exposed to a steady flow, there are two mechanisms about EC structural and functional integrity. In the first, normal laminar flow facilitates eNOS expression; it apparently suppresses TBP-2 expression and creates an anti-inflammatory milieu caused by the release of proinflammatory mediators such as TNF-*α* or IL-1*β* [16, 17, 66, 72]. In the second mechanism, TBP-2 expression is low, which reduces TBP-2-SHP2 interaction and leads to the dephosphorylation of CSK (making it inactive) and to the decrease of Src Y527 phosphorylation. Subsequently, Src is activated. Generally speaking, Src activation correlates with increased F-actin stress fiber formation. Stress fiber formation is vital for diverse cellular functions, such as migration, permeability, apoptosis, shape change, alignment, mechanosignal transduction, EC cell-cell junctions, and EC structural and functional integrity [66].

In contrast, a nonlaminar and low flow increases TBP-2 expression and the formation of vascular intercellular cell adhesion molecules (VCAMs), leading to the increased recruitment of leukocyte adhesions [16, 72]. It is believed that TBP-2 represses Kruppel-like factor 2, an important anti-inflammatory transcription factor induced by steady flow, although the exact interaction is unknown [72]. TBP-2 also regulates a SHP2-CSK-Src signaling cascade. Under disturbed flow conditions, TBP-2 expression is high, and CSK is active, leading to increased Src Y527 phosphorylation and low Src activity.

Finally, one study showed that the mechanism by which TBP-2 regulates mitochondrial metabolism is unclear, but it has been proposed that TBP-2 may regulate pyruvate dehydrogenase, thus promoting TCA cycle flux and oxidative phosphorylation. Interestingly, despite the fact that the deletion of TBP-2 could facilitate mitochondrial function in reducing ischemia-reperfusion damage, it has been verified that TBP-2 enhances the destruction of the myocardium, generated by ischemia-reperfusion via weakening anaerobic metabolism (e.g., anaerobic glycolysis), which enlarges the infarct size after a reversible coronary ligation [2]. For both complementary effects in ischemia-reperfusion, it is unfortunate that the authors did not explain which effect plays a dominant role.

**Figure 5:**
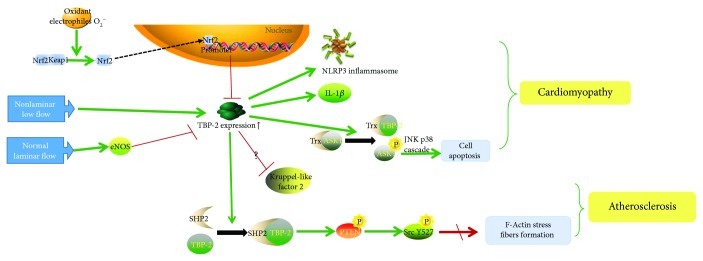
TBP-2 amplifies inflammatory response, apoptosis, and the reduction of stress fiber formation. Under oxidative stress, overexpressed TBP-2 results in the activation of the NLRP3 inflammasome, IL-1*β* and cell apoptosis and, ultimately, cardiomyopathy. Under a disturbed flow, overexpressed TBP-2 regulates a SHP2-CSK-Src signaling cascade, so that the cardiovascular system is susceptible to atherosclerosis.

To summarize, TBP-2 expression within the endothelium may amplify inflammatory response, apoptosis, the reduction of stress fiber formation, and the promotion of mitochondrial function (Figure 5). Whether under oxidative stress or disturbed flow, TBP-2 functions in the endothelial cells of the cardiovascular system to bring about cardiovascular cell death, structural weakness in vessels, cardiomyopathy, atherosclerosis, and ischemia-reperfusion damage.

### 4.4. TBP-2 in Lens Disease

Cataracts are the most common lens disease that can lead to blindness; it is the result of the cumulative long-term effect of multiple factors acting on the lens. Under oxidative stress reduced by ultraviolet rays (UV), radiation, or chemicals, ROS produced by mitochondria upsets the redox balance in the lens, so that the lens epithelial cells (LECs) die, and the proteins of the lens become dysfunctional.

TBP-2 and Trx act as central regulators of the cellular signaling pathways involved in the oxidative stress and apoptosis mechanisms. TBP-2 negatively regulates Trx function by binding with the reduced form of Trx and suppressing its activity [7]. Lou et al. [73] discovered that human lens epithelial (HLE-B3) cells induce a transient upregulation of Trx and a transient increase in Trx activity under H_2_O_2_ treatment. Subsequently, they demonstrated that TBP-2, which regulates Trx, is present in the LECs and makes the cells more susceptible to oxidative stress-induced apoptosis [74]. The Cys-247 of oxidized TBP-2 reacts with the Cys-32 of Trx to form an intermolecular disulfide bond at the redox-active catalytic domain, resulting in oxidative stress. A similar result (breaking of the Trx-ASK1 complex to activate apoptosis) was found in HLE-B3 cells [71].

For the apoptotic function of TBP-2, there is an approach that involves increasing the oxidation sensitivity, which leads to apoptosis. This process upregulates TBP-2 expression and the subsequent impairment of the thioredoxin antioxidative system through p38 MAPK in intracellular ROS. Afterwards, TBP-2 can separate Trx-ASK1 binding complexes and increase the Bax/Bcl-2 ratio and caspase3/7 activity to activate the ASK1 death pathway. It is worth mentioning that TBP-2 breaks not only the cytosolic Trx1-ASK1 complex but also the mitochondrial Trx2-ASK1 complex. However, how TBP-2 translocates from the cytosol to the mitochondria has not been worked out and needs further investigation. In this pathway, ASK1 is phosphorylated into a form that activates p38 MAPK and JUN to induce apoptosis, so that the Trx antiapoptotic function is disrupted, and the cell cycle is stopped at the G_2_/M stage [71].

**Figure 6:**
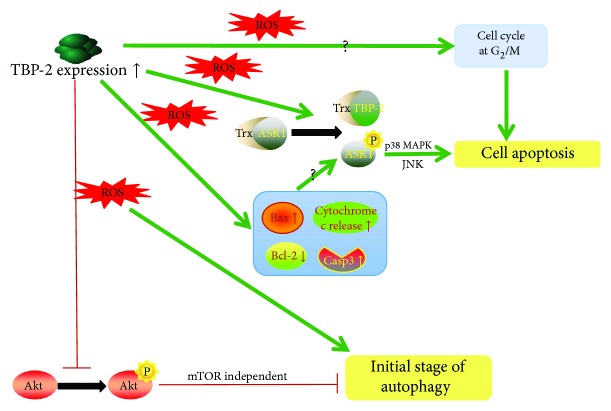
TBP-2 regulates autophagy and apoptosis of HLECs under oxidative stress. Under oxidative stress reduced by ROS, overexpressed TBP-2 can disrupt Trx-ASK1 binding complexes to suppress Trx bioavailability, and in the meantime, raise Bax/Bcl-2 ratio and caspase3/7 activity to activate the ASK1 death pathway to apoptosis. On the other hand, TBP-2 inhibits Akt/Bcl-xL signaling by mTOR independence to facilitate the initial stage of autophagy.

For the autophagic function of TBP-2, it performs a crucial function in the initial stage of autophagy by inhibiting Akt/Bcl-xL signaling, which is mTOR independent [3]. In general, LECs that overexpressed TBP-2 can suppress Akt/Bcl-xL signaling by mTOR independence. Research on autophagy in LECs and their signaling pathways under oxidative stress will provide a reliable experimental and research basis for preventing autophagy induced by ROS in LECs and will lead to further investigations of targeted therapies (Figure 6).

## 5. Conclusions and Future Directions

The studies reviewed here show that TBP-2 plays different roles in cancer, DM, cardiovascular disease, and cataracts. Whether TBP-2 is located in normal or abnormal tissues, it has the ability to kill cells in different ways, such as shortening the cell cycle or inducing apoptosis. With respect to the initial process of TBP-2 expression—namely, transcription—different TFs induced by the different stimulating factors combine with their corresponding binding sites or proteins to cause TBP-2 to overexpress. Upregulated TBP-2 expression performs many functions in different stages of life processes, such as proliferation, autophagy and apoptosis of cells, various kinds of metabolism, and inflammation. The most significant function of TBP-2 is that it can arrest the cell cycle at the G_0_/G_1_, G_1_/S, and G_2_/M phase transitions through the p38-JNK pathway in different cells and associate with Trx to inhibit Trx's ability to promote apoptosis. Consequently, cell growth is controlled.

Some questions remain about the detailed mechanisms of TBP-2 function in autophagy (including mitophagy) and cell cycle arrest. This review only shows that TBP-2 can promote the initial stage of autophagy which involves the phosphorylation of Akt, and although the mTOR-independent pathway has been mentioned in one study, the Akt-mTOR-dependent pathway—which plays a major role in autophagy—is not discussed in recent researches. We have already known how TBP-2 arrests the cell cycle at the G_0_/G_1_ and G_1_/S phase transitions, although the cell cycle arrests at G_2_/M has not yet been clarified.

Thus, more research is needed on the cell signaling pathways of autophagy that is generated by TBP-2. Moreover, the fact that TBP-2 acts as an antitumor gene and a facilitator of cell death could be utilized to develop novel strategies for the treatment of cancer and diabetes.
